# The Person-Event Data Environment: leveraging big data for studies of psychological strengths in soldiers

**DOI:** 10.3389/fpsyg.2013.00934

**Published:** 2013-12-13

**Authors:** Loryana L. Vie, Kevin N. Griffith, Lawrence M. Scheier, Paul B. Lester, Martin E. P. Seligman

**Affiliations:** ^1^Positive Psychology Center, University of PennsylvaniaPhiladelphia, PA, USA; ^2^Research Facilitation Team, Army Analytics GroupMonterey, CA, USA

**Keywords:** big data, psychological strengths, cost analysis, healthcare utilization, personnel data

## Abstract

The Department of Defense (DoD) strives to efficiently manage the large volumes of administrative data collected and repurpose this information for research and analyses with policy implications. This need is especially present in the United States Army, which maintains numerous electronic databases with information on more than one million Active-Duty, Reserve, and National Guard soldiers, their family members, and Army civilian employees. The accumulation of vast amounts of digitized health, military service, and demographic data thus approaches, and may even exceed, traditional benchmarks for Big Data. Given the challenges of disseminating sensitive personal and health information, the Person-Event Data Environment (PDE) was created to unify disparate Army and DoD databases in a secure cloud-based enclave. This electronic repository serves the ultimate goal of achieving cost efficiencies in psychological and healthcare studies and provides a platform for collaboration among diverse scientists. This paper provides an overview of the uses of the PDE to perform command surveillance and policy analysis for Army leadership. The paper highlights the confluence of both economic and behavioral science perspectives elucidating empirically-based studies examining relations between psychological assets, health, and healthcare utilization. Specific examples explore the role of psychological assets in major cost drivers such as medical expenditures both during deployment and stateside, drug use, attrition from basic training, and low reenlistment rates. Through creation of the PDE, the Army and scientific community can now capitalize on the vast amounts of personnel, financial, medical, training and education, deployment, and security systems that influence Army-wide policies and procedures.

As the quantity of data collected on members of the Army community (soldiers, family members, and civilian employees) continues to increase, researchers and information technology professionals are faced with a daunting task; effective management and analysis of increasingly larger pools of information with potentially stagnant or dwindling resource levels. Since 2006, the Army has incrementally developed the Person-Event Data Environment (PDE), a secure, collaborative research environment, to warehouse and study health, military service, and demographic information that is regularly collected on Army Active-Duty, Reserve, and National Guard soldiers, their family members, and Army civilian employees. This unique resource enables vetted researchers worldwide to mine this trove of data with a scientific mindset that can answer questions benefitting both the Army and wider scientific community.

This paper examines three aspects of the PDE. The first section focuses on the operating environment that gave rise to the PDE, primarily emphasizing challenges researchers have faced when working with sensitive but unclassified Department of Defense (DoD) data. The second aspect focuses on the structural design and operational capabilities of the PDE. This discussion includes the PDE’s physical and logical structure, the data contained within, and the statistical tools that are made available to researchers. Special attention is given to data sources that are most applicable to studies of psychological functioning as well as soldier outcomes that have important cost implications for the Army. The third section focuses on the types of research that may be conducted within the PDE. Where relevant, the authors have cited active research projects and multi-site collaborations. The paper concludes with a discussion of how the PDE can inform both science and policy.

## OPERATING ENVIRONMENT

The PDE was created to address several challenges that researchers face when working with DoD health, military service, and demographic data. These challenges include centralization of disparate data sources into an electronic repository, providing effective data security, and making the data accessible to researchers worldwide. The subsections below address each challenge in turn and how they have influenced the PDE’s development.

### BIG DATA OVERVIEW

The DoD digitizes an increasing amount of soldier information every day. Within the DoD, the Defense Manpower Data Center warehouses the largest archive of soldier performance data covering more than 43 million soldiers, veterans, their family members, and DoD civilian personnel (Defense Manpower Data Center Overview, 2012). The scope and size of these records are both steadily increasing over time as additional data elements are added and tracked. The accumulation of trillions of cells of information approaches, and may even exceed, traditional benchmarks for Big Data.

### CHALLENGES WITH DATA ACCESS

The sheer existence of such a repository does not necessarily imply that the data are accessible. The Army faces the same challenges that many large organizations face – stove-piped data collected for a specific purpose but not easily repurposed; specific policies that intentionally protect privacy, restrict access, and specify uses; and hesitancy to form collaborative relationships with outsiders (Landsbergen and Wolken, 2002). With this in mind, the burden falls on individual researchers to formulate data use agreements with each data source provider. This is, at best, a tedious and lengthy process and in other instances, this can represent an insurmountable barrier to research, particularly if a data asset provider resists sharing their data. The PDE aids in this effort by making as much data as possible available without the need for project-specific data use agreements. Furthermore, data providers are becoming increasingly familiar with the PDE and previously vetted, standardized data use agreement contract language may be used to help expedite the data request process.

### SECURITY OF DATA

The accumulation of large pools of data provides countless research possibilities and necessitates proper governance to prevent abuses and privacy violations (United States Government Accountability Office, 2008). For example, one can easily imagine a scenario in which a data-mining application classifies individuals on some basis of risk, grouping them into unique clusters, for instance, based on higher risk for suicide, drug use, violent crime, or some other sensitive objective criteria. Although individuals may be assigned into these higher risk groups, the assignment method will include a number of false positives due to the low base-rate of these outcomes. An accidental or intentional release of these classification results could have devastating effects on an individual’s career or social standing even though no transgression of Army regulations has been (or may ever be) committed. The PDE incorporates several security and governance features to maximize data security and prevent harm to study subjects.

## UNDER THE HOOD

The PDE is a secure, cloud-based research environment that provides data and software as a service. This section briefly describes the PDE system architecture, continues with a discussion of features that enhance privacy and security, and closes with an overview of the data and tools that are currently contained within the PDE.

### SYSTEM AND DATA ARCHITECTURE

**FIGURE 1:**
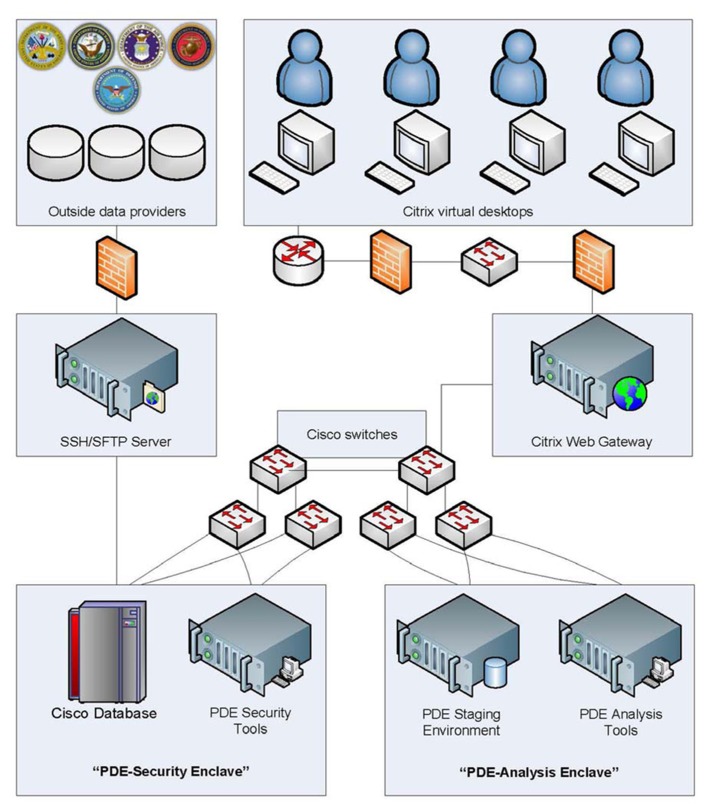
**PDE system architecture**.

**Figure 1** illustrates the separate security and analysis enclaves within the PDE. Only PDE administrative staff has access to the PDE-Security Enclave, a back-end server that houses the full data resource holdings. Data is not transferred from the PDE-Security Enclave until researchers submit and receive Federal approval for a study. At this time, data (acquired from a military data source provider) is then pushed to the PDE-Analysis Enclave, which researchers can access through a remote, secure connection. The architecture detailed in **Figure 1** incorporates numerous security and privacy features to protect the data, and these features are described below.

### SECURITY AND PRIVACY FEATURES

The traditional academic research model requires agencies to provide data to researchers. This creates duplication as many “ivory tower” researchers tend to work independently without drawing on shared institutional and personnel resources, let alone common data archives. Alternatively, the PDE brings researchers to the data. This is achieved through Citrix XenDesktop software, which allows users to securely access the data and software through an Advanced Encryption Standard 256-bit connection. Researchers are required to download and install a free copy of Citrix Receiver in order to use this service.

Data loaded into the PDE may not be extracted in any shape or form by researchers. Physical hardware and software limitations are in place to prevent breach of security protocols (i.e., data loss), such as no inter- or intra-net connections and no ability to copy and paste from the PDE to a user’s desktop. Users may request extraction of certain allowable non-data file types (e.g., Microsoft Word^®^ documents), at which point PDE staff will conduct a compliance review to ensure the requested files do not contain personally identifiable or protected health information. A similar vetting process is in place for uploading program or syntax files into the PDE.

**FIGURE 2:**
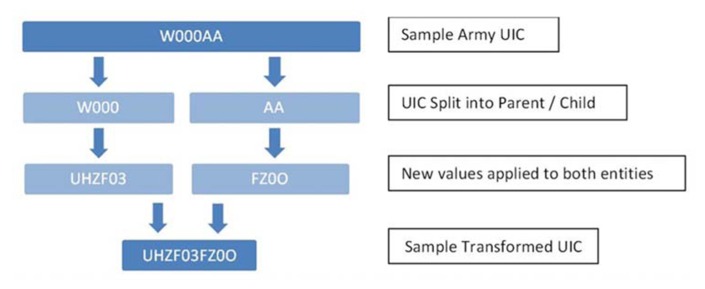
**Unit identification code (UIC) transformation**.

Personally identifiable and protected health information is securely transformed in order to minimize the risk of identifying individuals. Social security numbers (used to merge soldiers across time and data sources) are replaced with a random string of 12 digits that are unique to each individual and to each study. The algorithm “key” mapping this transformation is destroyed at the conclusion of the study. In addition to replacing social security numbers with a scrambled identifier, additional transformations of other personal identifiers are performed. This further reduces the risk of an individual being identified by an analyst, while maintaining enough information for standard analysis and reporting of aggregate data. For example, ranks and pay grades are condensed into groups, in some cases Julian dates are converted into YYYYMM format (or recorded only as YYYY), and unit identification codes are also scrambled. **Figure 2** diagrams the PDE procedure for transforming unit identification codes.

### REGULATORY FEATURES

Regulatory and compliance oversight of the PDE is provided by the Army Human Research Protections Office, the Army’s in-house equivalent of an “Institutional Review Board.” The Army Human Research Protections Office provides assurances that cover protection of the rights, welfare, and well-being for human subject research conducted or supported by the U.S. Army. These assurances are guided by the ethical principles set forth in the Belmont Report (Ethical Principles and Guidelines for the Protection of Human Subjects of Research) and the minimum standards set by the Department of Health and Human Services, Office for Human Research Protection regulations (45 CFR 46). In addition, Army Human Research Protections Office staff members conduct periodic assessments of current research projects to monitor compliance. More extensive compliance assurances are required for use of personally identifiable and protected health information involving medical data and covered under the standard Privacy Act regulations (Pub. L. No. 93-579) and subsequent amendments (USC Sec. 552a, Title 5, Part I, Chapter 5, Subchapter II). These assurances are further augmented by the Army human subjects policy provided in DoD 6025.18-R (DoD Health Information Privacy Regulation). Additionally, PDE staff members facilitate research by conducting “first-pass” reviews of proposals and assisting researchers with compliance questions and documentation.

### STATISTICAL TOOLS

The PDE remote desktop contains a suite of statistical, computational, and word processing tools that are made available to researchers. Currently these include Stata^®^, SPSS^®^, Mplus^®^, SAS^®^ Enterprise Guide^®^, Toad^TM^ for Oracle^®^, Qlikview^®^, R, and the Microsoft Office^®^ suite. Thus, in addition to extensive statistical modeling of Army data, analysis findings can be compiled for publication (peer-review articles or technical reports) entirely within the PDE. Some software packages have a limited number of “seat” licenses available, while others such as the freely available software program, R, allow unlimited use. Additional researcher-provided software and licenses may be loaded into the PDE if the software has been issued a Certificate of Networthiness from the Army Networthiness Program. The certificate procedures are designed to protect government information systems from security threats posed by commercial and other types of enterprise software. The Army’s current approved software list is not available on the web, but is publicly available through the PDE staff.

### DATA AVAILABILITY

The PDE’s data holdings are large and continually growing as new data assets are loaded. The holdings, currently totaling more than six terabytes (i.e., 6,000 gigabytes), are expected to double over the coming months. Currently, the PDE contains Army data on corrections and legal issues, physical fitness tests, military service information, deployments, demographics, training records, health assessments, medical visits, medical evacuations, injuries, deaths, psychological functioning, accessions, recruiting waivers, pay, and educational attainment. In the ensuing discussion we highlight datasets that can help illuminate the role of psychological assets in major cost drivers such as medical expenditures during deployment and stateside, drug use, attrition from basic training, and low reenlistment rates.

The Global Assessment Tool (GAT) is a primary tool used to assess psychosocial functioning in soldiers. Launched Army-wide in 2009, the GAT is a computerized, self-report questionnaire taken annually by all soldiers as part of the Comprehensive Soldier and Family Fitness (CSF2) program (Fravell et al., 2011). Soldiers may take the GAT up to four times a year, if desired. Upon completing the GAT, soldiers are provided normative feedback (based on a peer equivalent comparison group) on their relative strengths and weaknesses across the various psychosocial fitness dimensions. Then, as part of the CSF2 program, soldiers receive personalized recommendations for web-based trainings designed to further develop their psychological fitness. Since its inception, the GAT has been taken over 3.2 million times by over 1.3 million unique individuals (Office of CSF2, personal communication, August 26, 2013). The GAT assesses a range of psychological strengths, including self-management and coping skills, flexible thinking, positive affect, meaning, optimism, character strengths, social support, and social engagement, to name a few (Peterson et al., 2011).

Medical expenditures are major drivers of avoidable personnel costs for the U.S. Army. Tricare Management Activity is the health insurance provider for Active-Duty, National Guard, and Reserve soldiers, their families, survivors, and other eligible individuals. Tricare’s data holdings represent the largest collection of electronic medical records for the military. The Medical Data Repository is a Tricare-managed database housing electronic health records for all aspects of the medical treatment process conducted stateside. The repository includes medical encounters (e.g., physician office visits), surgical procedures, prescription use, and illness diagnoses (ICD-9 codes), to list a few examples. In addition, the repository houses a number of cost variables that are familiar to health economists, such as relative value units and medical expense performance and reporting system codes. Tricare’s Theater Medical Data Store maintains similar health records for services rendered while soldiers are deployed, although cost variables are not captured (but can be computed from standard economic healthcare valuation tables). Additionally, the quality and timeliness of the Theater Medical Data Store records varies as a function of the deployment environment in which the medical services are provided. For example, some medical services may be conducted informally during deployments and, as a result, are never formally documented in the data system.

Drug and alcohol abuse are also cost drivers for the Army. Costs due to substance use are not limited to direct medical care; costs are also incurred due to disciplinary action, outpatient treatment and prevention programs such as the Army Substance Abuse Program, and discharges from the Army. Furthermore, past research has demonstrated a link between psychological functioning and substance abuse. As a group, soldiers with positive urinalysis or breathalyzer tests for illicit drug use score significantly lower on the GAT survey instrument, even after controlling for demographic differences (Lester et al., 2011a). The Medical Data Repository and Theater Medical Data Store contain records on soldiers’ doctor visits and hospital services related to drug and alcohol abuse, but not all substance abuse problems result in medical treatment. Researchers may also request data from the Army’s Drug and Alcohol Management Information System, which tracks positive breathalyzer and urinalysis drug tests. Additionally, the Drug and Alcohol Management Information System captures when a unit commander makes a referral for a soldier to seek treatment and whether behavioral consultation referrals are consummated.

Attrition represents another major driver of personnel costs for the Army. Information pertaining to recruits who do not complete basic training is stored in the Medical Entrance Processing Command database, accessible through the PDE. After completing basic training, thousands of soldiers leave the Army or do not reenlist for a second term at the end of their military service contract. Information on soldiers who successfully complete basic training, and then subsequently attrit from the Army, is tracked in “Transaction Files” by the Defense Manpower Data Center, which are also accessible through the PDE.

Many of these assets are still only available to fully vetted PDE users with valid data use agreements. However, researchers are currently able to request access to military service information, deployments, demographics, accessions, pay, and waivers without a data use agreement. The PDE staff continues to work toward wider data availability, which is expected to grow as data providers become more comfortable with the PDE.

## RESEARCH FACILITATED BY THE PDE

This section describes three research efforts currently utilizing the strengths of the PDE. The first is a series of analyses conducted in support of the CSF2 mission, which evaluate the psychometric properties of the GAT and the efficacy of the CSF2 program. Next, we briefly discuss military-civilian collaboration between the Army, the University of Pennsylvania, and the Robert Wood Johnson Foundation. This breakthrough project seeks to identify positive psychosocial assets that enhance mental and physical well-being among U.S. Army soldiers. The third project represents collaboration between the Naval Postgraduate School and the Research Facilitation Team, which seeks to determine the cost implications of poor psychological health to the U.S. Army.

### U.S. ARMY COMPREHENSIVE SOLDIER AND FAMILY FITNESS

In support of CSF2, a series of research efforts have examined the normative relations between resilience (i.e., psychological health) and a wide range of negative and positive outcomes. For example, one line of inquiry has found that soldiers who receive low GAT scores have a significantly higher propensity for suicide, psychological illness, drug use, and criminal behavior (Lester et al., 2011a; Harms et al., 2013). A different line of inquiry has linked higher GAT scores to accelerated promotions, promotion to brigadier general, selections for command assignments, and Army career fields that require professional degrees (Lester et al., 2011b).

Additionally, scientists have evaluated the Master Resilience Training (MRT) program, a “train the trainer” intervention developed to foster psychological resilience in soldiers. Exposure to MRT was associated with statistically significant increases in resilience and psychological health, and a slightly lower likelihood (11% decrease, based on the corresponding odds ratio) of being diagnosed with a mental health problem, such as post-traumatic stress disorder (PTSD; Harms et al., 2013). Furthermore, soldiers exposed to the MRT program displayed a 58% lower rate of substance abuse problem diagnoses (based on the corresponding odds ratio); significantly lower than their counterparts who were not exposed to MRT. The PDE was instrumental in facilitating access to the MRT program evaluation data assets. Furthermore, consistent with the instrumental goals of the PDE, the corresponding reductions in mental health and substance abuse problem diagnoses could represent tremendous cost-saving potential for the Army.

### MILITARY-CIVILIAN COLLABORATION

In 2011, the Robert Wood Johnson Foundation awarded the University of Pennsylvania (Penn) a grant to examine predictors of positive health in the Army. A major component of this grant revolved around creating a foundation for future military-civilian collaboration. Toward this end, the Penn team began by re-examining the psychometric structure of the GAT. Confirmatory factor analysis models posited a lower-order factor structure including positive cognitions, self-management skills, positivity (affect), character strengths (e.g., warmth), and social engagement (e.g., social network; Vie et al., 2013). In addition, a higher-order model of psychological strengths adequately accounted for the moderate statistical relations among the primary factors. This more refined and parsimonious representation of GAT strengths enables more precise examination of predictors of positive health and is well suited for examining criterion-related validity. A chronometric model demonstrated that psychological assets increase in a linear and positive manner over time. Subsequent analyses have also conditioned growth in psychological assets on various deployment indices and demographic factors (e.g., gender, age). In addition, measurement invariance is being tested across various demographic subgroups, in order to identify whether GAT subscales have unique meaning for different subgroups (e.g., gender, age, education, and marital status).

The Penn team is currently studying the impact of combat deployments on psychological health. Deployment represents an important, and highly relevant, potentially stressful event that many Army soldiers experience at some point during their service. Past research has examined development of PTSD symptoms following deployment. However, rather than taking a deficit approach (i.e., assessing ill-being), Penn researchers are capitalizing on the data resources in the PDE by examining associations between deployment experiences and soldiers’ levels of psychosocial strengths over time. The team is also examining relations between pre-deployment psychosocial strengths and post-deployment depressive symptoms and negative affect. Preliminary analyses reveal that having fewer psychosocial strengths is associated with more depressive symptoms and negative affect post-deployment (controlling for early symptom levels), whereas having more psychosocial strengths is protective and associated with fewer depressive symptoms and less negative affect post-deployment.

### ECONOMIC ANALYSES

Economists at the Naval Postgraduate School are working to determine the costs of poor resilience and psychological health to the Army. In addition, efforts are also underway to identify the potential economic benefits that could accrue to the Army through recruiting, training, and maintaining a psychologically healthy and resilient force. Poor psychological functioning places a significant burden on the Army (e.g., through costs related to attrition, healthcare utilization, and psychological illness), and the struggle for armed forces mental health professionals to treat illnesses following combat has been considered by some the Army’s “third front” (Thompson, 2010). On this issue, Former Defense Secretary Robert Gates once declared, “health care costs are eating the Defense Department alive” (Gates, 2010). The initial phase of this research effort emphasizes four cost elements related to poor psychological functioning – attrition during initial military training, attrition before completion of first-term enlistments, increased medical usage during deployments, and a higher likelihood of receiving a diagnosis for PTSD or major depression post-deployment. Preliminary results have demonstrated a significantly higher risk of attrition for recruits that are in the bottom 10% of GAT scores, the latter treated as four dimensions assessing spiritual, emotional, social, and family fitness. Furthermore, the results include the first-ever estimate of the annual costs to the Army and DoD for treatment of PSTD and major depression. Future phases may include additional cost elements such as increased usage of medical care stateside, heightened risk of drug and alcohol abuse, increased likelihood of committing a criminal offense, and other negative soldier outcomes.

Estimates of annual Army expenditures related to the treatment of PTSD alone range from $1.54 to $2.69 billion (Cesur et al., 2011). However, these are merely estimates based on either simulations or extrapolated treatment costs obtained from civilian medical facilities. The current research program utilizes actual patient encounter data provided by Tricare to determine the actual annual expenditures that are directly attributable to PTSD and major depression. Additionally, costs of basic training are estimated at $73,000 per soldier (U.S. Army Recruiting Command, 2011). Considering that tens of thousands of Army recruits attrit during basic training and thousands more soldiers attrit before completion of their first-term enlistments, the cost of attrition quickly escalates into hundreds of millions of dollars per annum. Even small reductions in the attrition rate could result in potential savings of tens of millions of dollars each year.

Once completed, the findings from this project will enable decision-makers to determine the potential savings to the Army and DoD through increases in force-wide psychological health. Currently, researchers may only observe the costs of behavioral health programs and the subsequent changes in soldier outcomes, without knowing the true monetary value of any positive changes observed. These savings, once known, allow for more inclusive program evaluation that includes cost-benefit analysis of CSF2 or other behavioral health interventions. Military leaders responsible for making decisions regarding instituting force-wide training will then be able to observe the return on investment for such programs.

## DISCUSSION

The PDE is currently set to expand significantly in the near future. This expansion entails both procuring additional data assets and physical hardware to increase PDE bandwidth. Once fully operational, the PDE represents a tremendous opportunity for scientific advancement via research and careful analysis. Those working diligently toward meeting this goal often characterize the promise of the PDE as potential energy that, if properly focused, could lead to significant breakthroughs benefitting science and humanity. Promise aside, there are a few basic tenets that must hold true in order for the PDE to become the standard for the governance of, security for, access to, and analysis of Army and DoD data.

First, we must carefully attend to the notion that the purpose of the PDE is to bring scientists to the data, rather than pushing data to scientists. Put succinctly, the data in the PDE describe one of the U.S.’s most valuable resources – the men and women of our military community – so proper governance is warranted. Second, we must continue to expand the data held within the PDE in order to keep it relevant. While the PDE likely represents the broadest range of DoD personnel data, there are many other datasets that have yet to be integrated into the PDE; such integration will be iterative as new data emerge, and it will likely take years to bring in the lion’s share of existing data. Third, the PDE must be easy to use and continuously improve based on user feedback, if we expect the research community to utilize this platform. Finally, while we must be mindful of our security and privacy concerns, we must also trust our processes and governance systems if we are to meet our ultimate goal – providing access to the broader scientific community.

Keeping true to these tenets falls to the Army’s Research Facilitation Team. The Research Facilitation Team – whose staff consists of research psychologists, economists, and Big Data information technology experts – was established in 2013 to not only provide governance over the PDE, but also to assist researchers in using the PDE. Given the metadata needed to navigate the vast data holdings within the PDE, this team is invaluable in shepherding new users and their projects through the data request, review, and analysis process. The Research Facilitation Team fosters collaboration within the PDE user community so that participating government organizations and researchers may survey the wide variety of work done within the PDE. This is all done with an eye toward leveraging efficiencies and reducing redundancies across research projects.

The PDE represents a rare opportunity to connect the military with the wealth of knowledge and experience contained within the wider scientific community. Working side-by-side, military and academic researchers will collaborate and examine the confluence of psychological health, soldier performance, economics, and more. The results of this research may inform Army-wide policy decisions regarding recruitment, prevention and treatment programs, job assignments, manpower training, and budgeting. The PDE represents a growing opportunity to unify science, data, and technology under one umbrella in order to bring the best information to bear on issues which have widespread implications for the DoD.

## Conflict of Interest Statement

The authors declare that the research was conducted in the absence of any commercial or financial relationships that could be construed as a potential conflict of interest.
